# FAIR Island: real-world examples of place-based open science

**DOI:** 10.1093/gigascience/giad004

**Published:** 2023-03-20

**Authors:** Erin Robinson, Matthew Buys, John Chodacki, Kristian Garzas, Steven Monfort, Catherine Nancarrow, Maria Praetzellis, Brian Riley, Sarala Wimalaratne, Neil Davies

**Affiliations:** Metadata Game Changers, LLC, University of Colorado, Boulder, CO 80304, USA; DataCite, Hannover 30167, Germany; California Digital Library, University of California, Office of the President, Oakland, CA 94607, USA; DataCite, Hannover 30167, Germany; Natural Reserve System, Office of Research & Innovation, University of California, Oakland, CA 94607, USA; California Digital Library, University of California, Office of the President, Oakland, CA 94607, USA; California Digital Library, University of California, Office of the President, Oakland, CA 94607, USA; California Digital Library, University of California, Office of the President, Oakland, CA 94607, USA; DataCite, Hannover 30167, Germany; Gump South Pacific Research Station, University of California, Moorea 98728, French Polynesia, and Berkeley Institute for Data Science, University of California, Berkeley, CA 94720, USA

**Keywords:** place-based research, FAIR Principles, CARE Principles, research data infrastructure, data policy, data management plans

## Abstract

The relationship between people, place, and data presents challenges and opportunities for science and society. While there has been general enthusiasm for and work toward Findable, Accessible, Interoperable, and Reusable (FAIR) data for open science, only more recently have these data-centric principles been extended into dimensions important to people and place—notably, the CARE Principles for Indigenous Data Governance, which affect collective benefit, authority to control, responsibility, and ethics. The FAIR Island project seeks to translate these ideals into practice, leveraging the institutional infrastructure provided by scientific field stations. Starting with field stations in French Polynesia as key use cases that are exceptionally well connected to international research networks, FAIR Island builds interoperability between different components of critical research infrastructure, helping connect these to societal benefit areas. The goal is not only to increase reuse of scientific data and the awareness of work happening at the field stations but more generally to accelerate place-based research for sustainable development. FAIR Island works reflexively, aiming to scale horizontally through networks of field stations and to serve as a model for other sites of intensive long-term scientific study.

## Background

Relationships between human communities and their natural and built environments are increasingly mediated through digital data. These data feed models and algorithms that impact decision-making in a range of contexts and at nested scales of governance, from the stewardship of smart cities and Indigenous lands to international agreements over global commons such as the high seas, Antarctica, or the Earth's atmosphere. Digital representations of these complex systems (digital twins or avatars) are emerging as technology platforms that harness the predictive power of scientific understanding (e.g., the consequences of climate change), while raising vital ethical, legal, and social issues, including who should control these capabilities and how.

**Figure 1:**
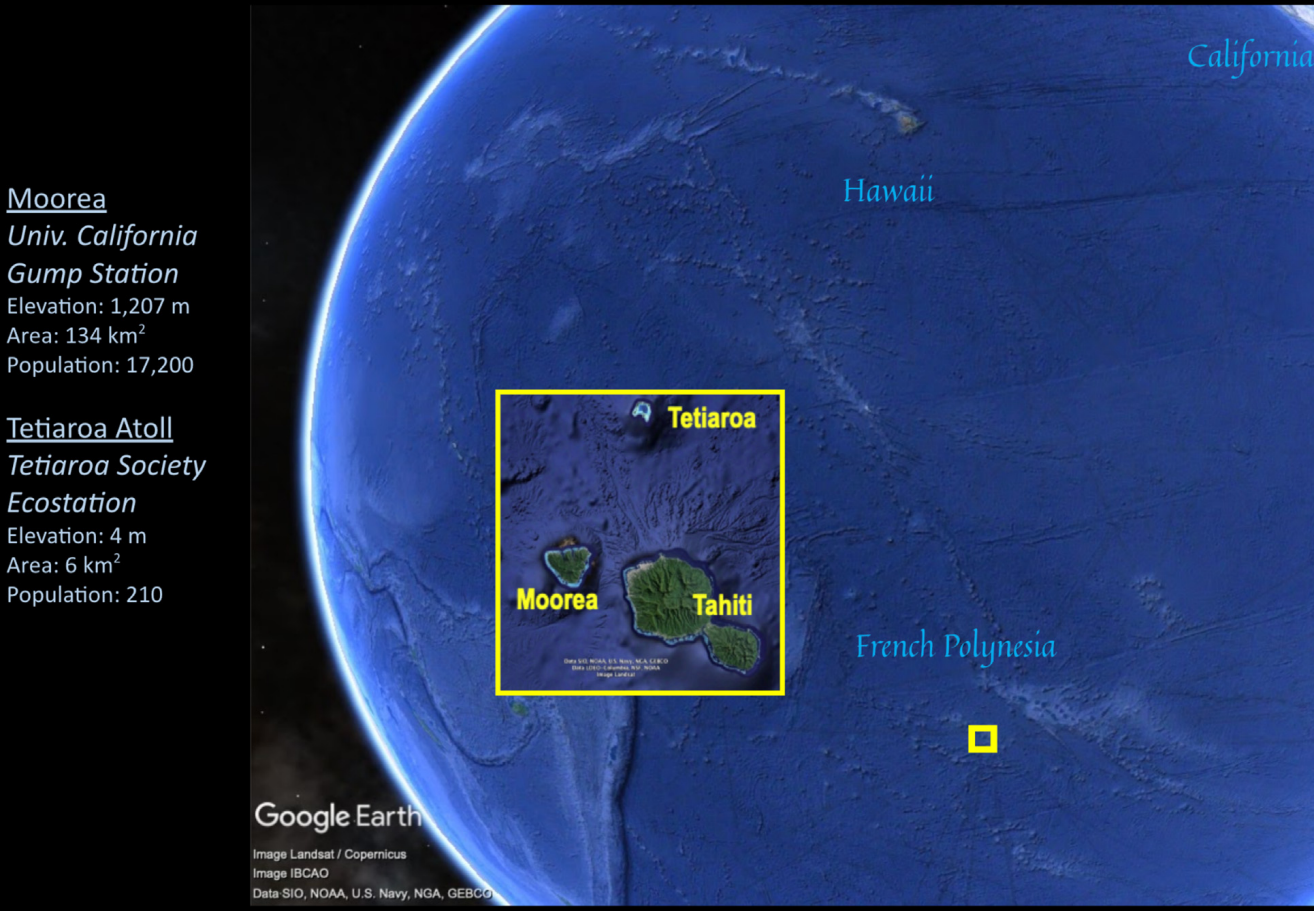
Map of first 2 field station site locations.

Many aspirations for more effective data sharing in science are situated at a high level, such as the Beijing Declaration on Research Data [1], while principles such as the Findable, Accessible, Interoperable, and Reusable (FAIR) data principles and the CARE (Collective benefit, Authority to control, Responsibility, Ethics) principles for Indigenous data governance [2, 3] have gained significant traction. Real-world implementations are now needed to demonstrate appreciable scientific and societal benefits. For example, do FAIR and CARE enhance the capacity to integrate diverse data types in the transdisciplinary predictive modeling of complex social-ecological systems [4] that underpins sustainability science? The FAIR Island project seeks to address these questions, leveraging networks of field stations and marine laboratories as study systems. We start with networks in (i) French Polynesia, including the University of California (UC) Gump South Pacific Research Station on Moorea and a recently established research station on the atoll of Tetiaroa (Fig.   1), and (ii) California, through the UC Natural Reserve System (UCNRS), which operates 41 stations across the state. FAIR Island draws on significant data expertise provided by the Berkeley Institute for Data Science, the Island Digital Ecosystem Avatars (IDEA) Consortium, the California Digital Library (CDL), DataCite, and Metadata Game Changers LLC.

## FAIR Island Approach

**Figure 2:**
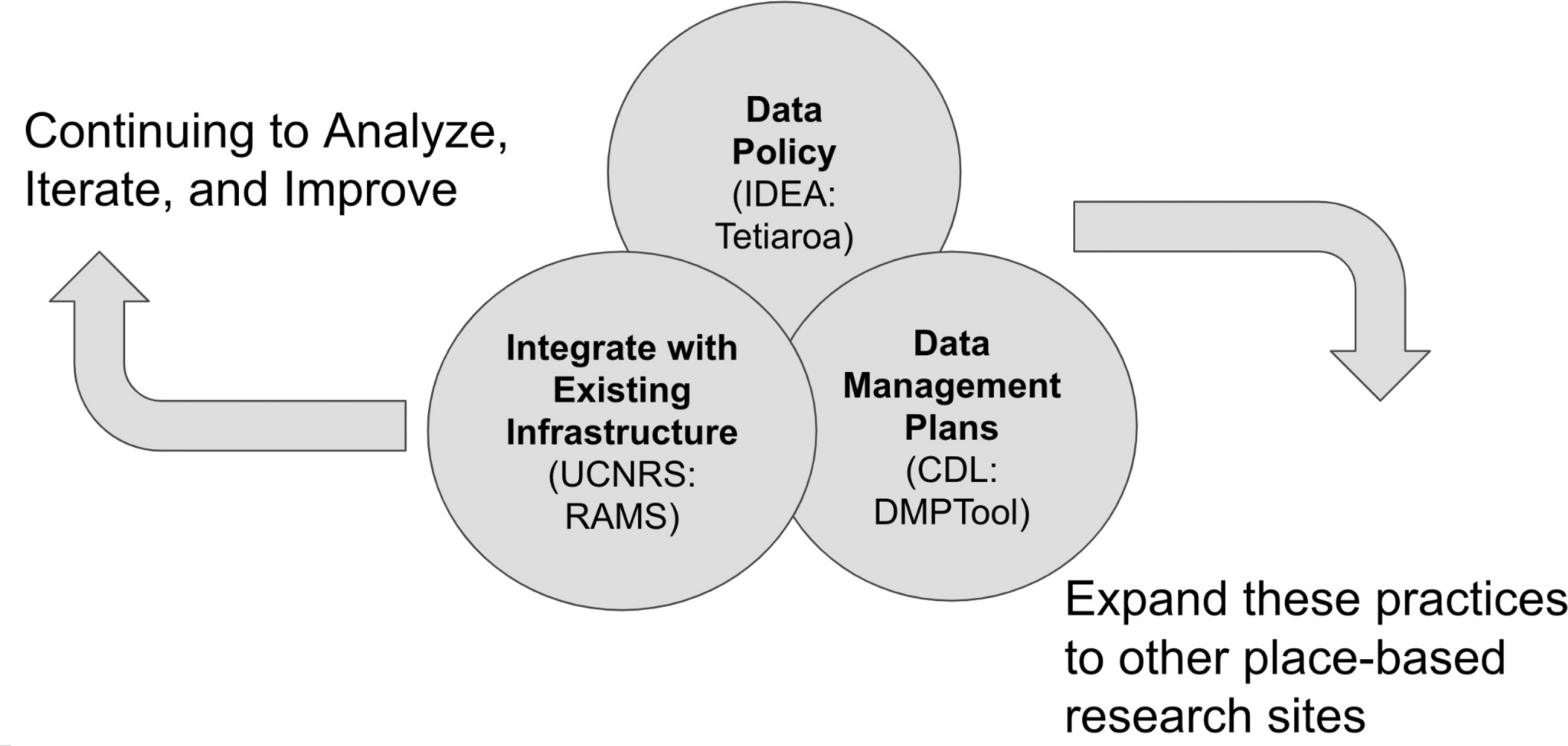
FAIR Island project diagram.

Launched in 2019 with seed funding from the University of California, FAIR Island received additional support from the National Science Foundation in August 2021. The project focuses initially on two working field stations—one new and one well established—that are linked to a range of international research networks in marine and terrestrial environments. Informed by experience at the Gump Station (established in 1985), the new international research station (Tetiaroa Ecostation) on the private island of Tetiaroa offers an opportunity to build optimal data infrastructure and practices from the ground up and to demonstrate the benefits for all stakeholders. Drawing on real-use cases, FAIR Island contributes to the advancement and adoption of open science at the field stations by building interoperability between existing tools and infrastructure, including data management plans (DMPs), research practice, persistent identifiers (PIDs), data policy, and publications (Fig.   2). In addition to a scientific and data-centric focus, the project also addresses ethical, legal, and social issues through application of the CARE principles [3]. We believe that improving research data infrastructure and practice across these scientific, ethical, legal, and social dimensions at field stations will accelerate place-based research to solve global challenges and benefit local communities (e.g., achieving UN Sustainable Development Goals). Once the FAIR Island infrastructure is established at the initial two stations, the common elements will be shared with other field stations in the Pacific islands [ 5], California, and beyond. Through these efforts, we will integrate and test the FAIR and CARE principles addressing whether they have synergies or trade-offs and the extent to which their implementation has measurable impacts on community acceptance, reuse of data, traceability of data products, and rewards/credit for compliance with the principles.

### Develop and test place-based research data policies

At the time of writing, the project has collected existing data policy resources, developed a draft Data Policy for the Tetiaroa Ecostation, and solicited initial feedback from the data curation community, including participating in the FAIRsFAIR data policy evaluation [6], and is beginning to get feedback from the stakeholders on Tetiaroa, starting with Tetiaroa Society's Scientific and Cultural Advisory Boards. Building from the draft Tetiaroa Data Policy, we created and published a Generic Place-Based Data Policy [7] as a template to be shared, refined, and implemented across various field stations and marine labs. By using GitHub for version control, the development process for this policy is fully transparent, enabling others to easily adopt and adapt it for their use.

### Adapting user-driven systems integrations for networked DMPs

For more than 10 years, Data Management Plans (DMPs) have been included in the proposal phase of the research life cycle. FAIR Island now extends the use of the DMPs into the implementation phase of research by leveraging the DMPTool to establish a detailed research DMP for work at a field station and mint a digital object identifier (DOI) for that DMP. As research outputs occur (e.g., data, software, and publications), they are linked to the networked DMP through PIDs, creating a hub of activity about a particular research project.

Integration with other systems is a core requirement and scalable benefit of FAIR Island. These PID connections facilitate further development of the DataCite Commons interface and extend connections made possible via the networked DMP. Users can track relationships between DMPs, investigators, outputs, organizations, research methods, and protocols, as well as display citations throughout the research life cycle. A second integration with the UCNRS optimizes the UCNRS reservations management tool (Reservation Application Management System [RAMS]; https://rams.ucnrs.org/applications), which is used at many field stations and provides the initial project metadata needed to create a DMP. This integration reduces the burden on the researcher, who need not enter information twice.

**Figure 3:**
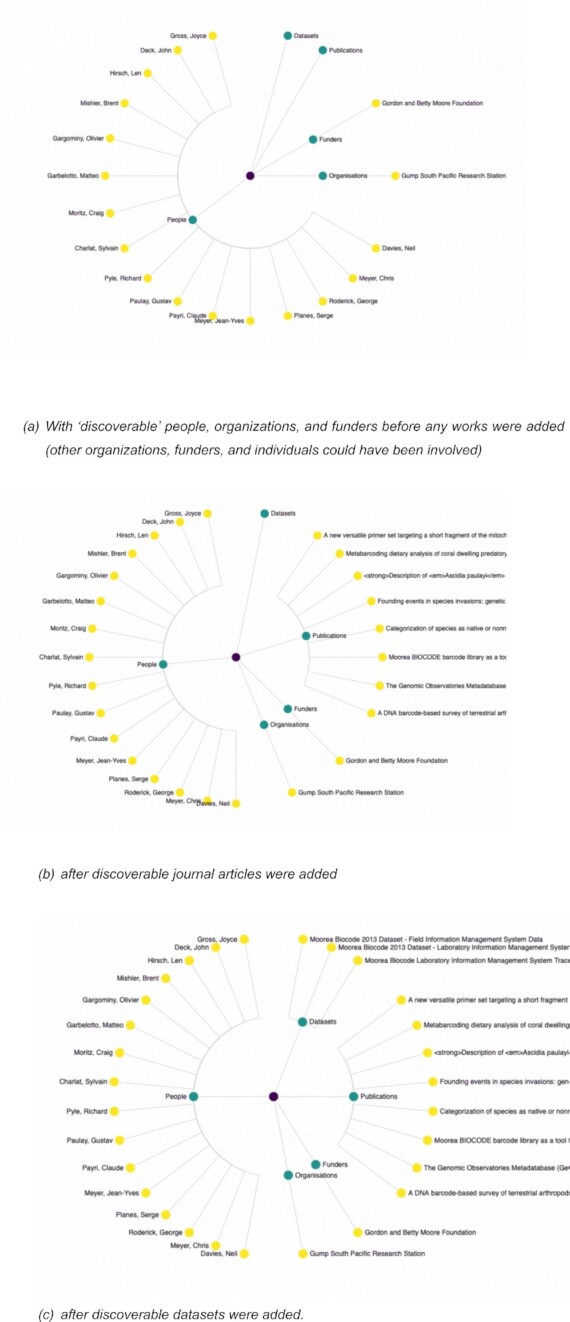
Moorea Biocode PID Graph Evolution [10] (a) before any works were added, (b) after journal articles were added, and (c) after datasets were added.

As an initial integration experiment, FAIR Island selected the Moorea Biocode Project [8], which ended about 10 years ago. We created a DMP (see https://dmphub.cdlib.org/dmps/doi:10.48321/D1F88S) using the DMPTool with content from the original application submitted to UCNRS RAMS. To find research outputs, the literature was searched for the term “Moorea Biocode.” Identified publications and datasets were then linked through related works in the DMP. With these links established, the DataCite DMP ID Jupyter Notebook [9] was used to draw 3 graphs (Fig. 3) that simulate the way that the PID Graph can visualize relationships across the research life cycle.

This initial experiment showed the benefits of having structured metadata that included PIDs and could be updated to include scholarly outputs like data and papers. We will ask that each research project have a DMP, and if not, we will require using the DMPTool to create one. Moving forward, we are experimenting with minting project DOIs for research projects based on their approved applications. Utilizing the DOI infrastructure will allow for the project metadata to be reused for other aspects of project administration (e.g., permits), reducing the researcher burden. It will also allow for the inclusion of related identifiers, like funding information, DMPs, or academic outputs, giving us the benefits found in our initial experiment.

Utilizing this collection of projects with connected outputs, we will develop a field station dashboard showing field data collection to publication, documenting all research data and research outcomes derived from those data. These linkages will provide insight into not only direct connections but also connections several steps removed, realizing the machine-actionable vision of the FAIR Principles. The understanding is that the dashboard will provide an easier way for field station staff to communicate the value of the work happening at these sites to various stakeholders, including funders and local resource managers. It will also facilitate reuse of data and other research outputs to conduct new science.

## Conclusions

The FAIR Island project is iteratively adjusting to the realities on the ground as it works with real-use cases at participating field stations. Each new implementation will allow us to further refine the research infrastructure. The outcomes of this project will provide insights on optimal research data management practices and how to support place-based research more effectively. The impacts of the project are likely to be far-reaching beyond field stations—to smart cities and other place-based research sites. For more on the project and to get involved, see the website (https://fairisland.org/).

## Abbreviations

CARE: Collective benefit, Authority to control, Responsibility, Ethics; CDL: California Digital Library; CODATA: Committee on Data for Science and Technology; DMP: Data Management Plan; DOI: digital object identifier; FAIR: Findable, Accessible, Interoperable, Reusable; IDEA: Island Digital Ecosystem Avatars; PID: persistent identifier; RAMS: Reservation Application Management System; UC: University of California; UCNRS: University of California Natural Reserve System.

## Supplementary Material

giad004_GIGA-D-22-00310_Original_Submission

giad004_GIGA-D-22-00310_Revision_1

giad004_Response_to_Reviewer_Comments_Original_Submission

giad004_Reviewer_1_Report_Original_Submission Larry Lannom, MLS -- 12/5/2022 Reviewed

giad004_Reviewer_2_Report_Original_Submission Timothy Clark, Ph.D. -- 12/13/2022 Reviewed

## Data Availability

The artifacts produced through the FAIR Island Project and reported on here are shared through the FAIR Island Zenodo community, https://zenodo.org/communities/fairisland
