# Supplementary material for: FAIR Island: real-world examples of place-based open science
Source: Gigascience. 2023 Mar 20;12:giad004. doi: 10.1093/gigascience/giad004 (PMC10025936; doi:10.1093/gigascience/giad004)
Supplement: giad004_GIGA-D-22-00310_Revision_1 [file giad004_giga-d-22-00310_revision_1.pdf]

GigaScience

FAIR Island: Real-World Examples of Place-based Open Science

--Manuscript Draft--

**Manuscript Number:**	GIGA-D-22-00310R1
**Full Title:**	FAIR Island: Real-World Examples of Place-based Open Science
**Article Type:**	Commentary
**Funding Information:**	National Science Foundation (2132549) — Mr John Chodacki<br>National Science Foundation (2129268) — Dr. Neil Davies
**Abstract:**	The relationship between people, place, and data presents challenges and opportunities for science and society. While there has been general enthusiasm for and work towards Findable, Accessible, Interoperable, and Reusable (FAIR) data for Open Science, only more recently have these data-centric principles been extended into dimensions important to people and place. Notably, the CARE Principles for Indigenous Data Governance, which affect collective benefit, authority to control, responsibility, and ethics. The FAIR Island Project seeks to translate these ideals into practice leveraging the institutional infrastructure provided by scientific field stations. Starting with field stations in French Polynesia as key use cases that are exceptionally well connected to international research networks, FAIR Island builds interoperability between different components of critical research infrastructure, helping connect these to societal benefit areas. The goal is not only to increase reuse of scientific data and the awareness of work happening at the field stations, but more generally to accelerate place-based research for sustainable development. FAIR Island works reflexively, aiming to scale horizontally through networks of field stations and to serve as a model for other sites of intensive long-term scientific study.
**Corresponding Author:**	Neil Davies, Ph.D.<br>University of California Berkeley<br>Berkeley, California UNITED STATES
**Corresponding Author Secondary Information:**	
**Corresponding Author's Institution:**	University of California Berkeley
**Corresponding Author's Secondary Institution:**	
**First Author:**	Erin Robinson
**First Author Secondary Information:**	
**Order of Authors:**	Erin Robinson<br>Matthew Buys<br>John Chodacki<br>Krisitan Garza<br>Steven Monfort<br>Catherine Nancarrow<br>Maria Praetzellis<br>Brian Riley<br>Sarala Wimalaratne<br>Neil Davies, Ph.D.
**Order of Authors Secondary Information:**	
**Response to Reviewers:**	We are grateful for the Reviewer's comments.

Powered by Editorial Manager® and ProduXion Manager® from Aries Systems Corporation

	In response to Reviewer #1 we have have revised the text to introduce the IDEA project earlier.<br><br>In response to Reviewer #2:<br><br>As requested, we have toned down the rhetoric and revised the text accordingly, indicating our overarching goal rather than presenting a hypothesis to test. We have also modified the Abstract accordingly.<br><br>We agree with framing the project as an attempt to test whether the FAIR and CARE principles lead to improvements and we have modified the text to reflect this better.<br><br>Further clarification was indeed needed on who we meant by "the community" and we have modified the text to clarify the current status of community feedback and our future intentions.
**Additional Information:**	
**Question**	**Response**
Are you submitting this manuscript to a special series or article collection?	No
**Experimental design and statistics**<br><br>Full details of the experimental design and statistical methods used should be given in the Methods section, as detailed in our Minimum Standards Reporting Checklist. Information essential to interpreting the data presented should be made available in the figure legends.<br><br>Have you included all the information requested in your manuscript?	Yes
**Resources**<br><br>A description of all resources used, including antibodies, cell lines, animals and software tools, with enough information to allow them to be uniquely identified, should be included in the Methods section. Authors are strongly encouraged to cite Research Resource Identifiers (RRIDs) for antibodies, model organisms and tools, where possible.<br><br>Have you included the information requested as detailed in our Minimum Standards Reporting Checklist?	Yes

Powered by Editorial Manager® and ProduXion Manager® from Aries Systems Corporation

**Availability of data and materials**<br><br>All datasets and code on which the conclusions of the paper rely must be either included in your submission or deposited in publicly available repositories (where available and ethically appropriate), referencing such data using a unique identifier in the references and in the “Availability of Data and Materials” section of your manuscript.<br><br>Have you have met the above requirement as detailed in our Minimum Standards Reporting Checklist?	Yes

Powered by Editorial Manager® and ProduXion Manager® from Aries Systems Corporation

Manuscript

Click here to access/download;Manuscript;Revision_FAIR Island Marker Paper.pdf

FAIR Island: Real-World Examples of Place-based Open Science

Erin Robinson, Metadata Game Changers, LLC, University of Colorado, Boulder CO, 80304, email: erin@metadatagamechangers.com

Matthew Buys, DataCite, Welfengarten 1 B, 30167 Hannover, email: mattbuys@datacite.org

John Chodacki, California Digital Library, University of California, Office of the President, 1111 Franklin Street, Oakland, CA 94607, email: John.Chodacki@ucop.edu

Kristian Garza, DataCite, Welfengarten 1 B, 30167 Hannover, email: kgarza@datacite.org

Steven Monfort, Natural Reserve System, Office of Research & Innovation, University of California System, 1111 Franklin Street, 11th Floor, Oakland, CA 94607-5200, email: steve.monfort@ucop.edu

Catherine Nancarrow, California Digital Library, University of California, Office of the President, 1111 Franklin Street, Oakland, CA 94607, email: catherine.nancarrow@ucop.edu

Maria Praetzellis, California Digital Library, University of California, Office of the President, 1111 Franklin Street, Oakland, CA 94607, email: Maria.Praetzellis@ucop.edu

Brian Riley, California Digital Library, University of California, Office of the President, 1111 Franklin Street, Oakland, CA 94607, email: brian.riley@ucop.edu

Sarala Wimalaratne, DataCite, Welfengarten 1 B, 30167 Hannover, email: sarala@datacite.org

Neil Davies, Gump South Pacific Research Station, University of California, BP 244, Moorea 98728, French Polynesia; Berkeley Institute for Data Science, University of California, 190 Doe Library, Berkeley, CA 94720, USA; E-mail: ndavies@berkeley.edu

Keywords

Place-based research, FAIR Principles, CARE Principles, research data infrastructure, data policy, data management plans

Abstract

The relationship between people, place, and data presents challenges and opportunities for science and society. While there has been general enthusiasm for and work towards Findable, Accessible, Interoperable, and Reusable (FAIR) data for Open Science, only more recently have these data-centric principles been extended into dimensions important to people and place. Notably, the CARE Principles for Indigenous Data Governance, which affect collective benefit, authority to control, responsibility, and ethics. The FAIR Island Project seeks to translate these ideals into practice leveraging the institutional infrastructure provided by scientific field stations. Starting with field stations in French Polynesia as key use cases that are exceptionally well connected to international research networks, FAIR Island builds interoperability between different components of critical research infrastructure, helping connect these to societal benefit areas. The goal is not only to increase reuse of scientific data and the awareness of work happening at the field stations, but more generally to accelerate place-based research for sustainable development. FAIR Island works reflexively, aiming to scale horizontally through networks of field stations and to serve as a model for other sites of intensive long-term scientific study.

Background

Relationships between human communities and their natural and built environments are increasingly mediated through digital data. These data feed models and algorithms that impact decision-making in a range of contexts and at nested scales of governance, from the stewardship of smart cities and Indigenous lands to international agreements over global commons such as the High Seas, Antarctica, or the Earth's atmosphere. Digital representations of these complex systems (digital twins or avatars) are emerging as technology platforms that harness the predictive power of scientific understanding (e.g., the consequences of climate change), while raising vital ethical, legal, and social issues, including who should control these capabilities and how.

Many aspirations for more effective data sharing in science are stated at a high level, such as the Beijing Declaration on Research Data [1], while principles such as the FAIR data principles and the CARE principles for Indigenous data governance [2,3] have also gained significant traction. Real-world implementations are now needed to demonstrate appreciable scientific and societal benefits. For example, do FAIR and CARE enhance the capacity to integrate diverse data types in the transdisciplinary predictive modeling of complex social-ecological systems [4], which underpins sustainability science? The FAIR Island project seeks to address these questions leveraging networks of field stations and marine laboratories as study systems. We start with networks in (a) French Polynesia, including the University of California (UC) Gump South Pacific Research Station on Moorea, and a recently established research station on the atoll of Tetiaroa (Fig 1), and (b) California, through the UC Natural Reserve System (UCNRS), which operates 41 stations across the state. FAIR Island draws on significant data expertise provided by the Berkeley Institute for Data Science, the Island Digital Ecosystem Avatars (IDEA) Consortium, the California Digital Library (CDL), DataCite, and Metadata Game Changers LLC.

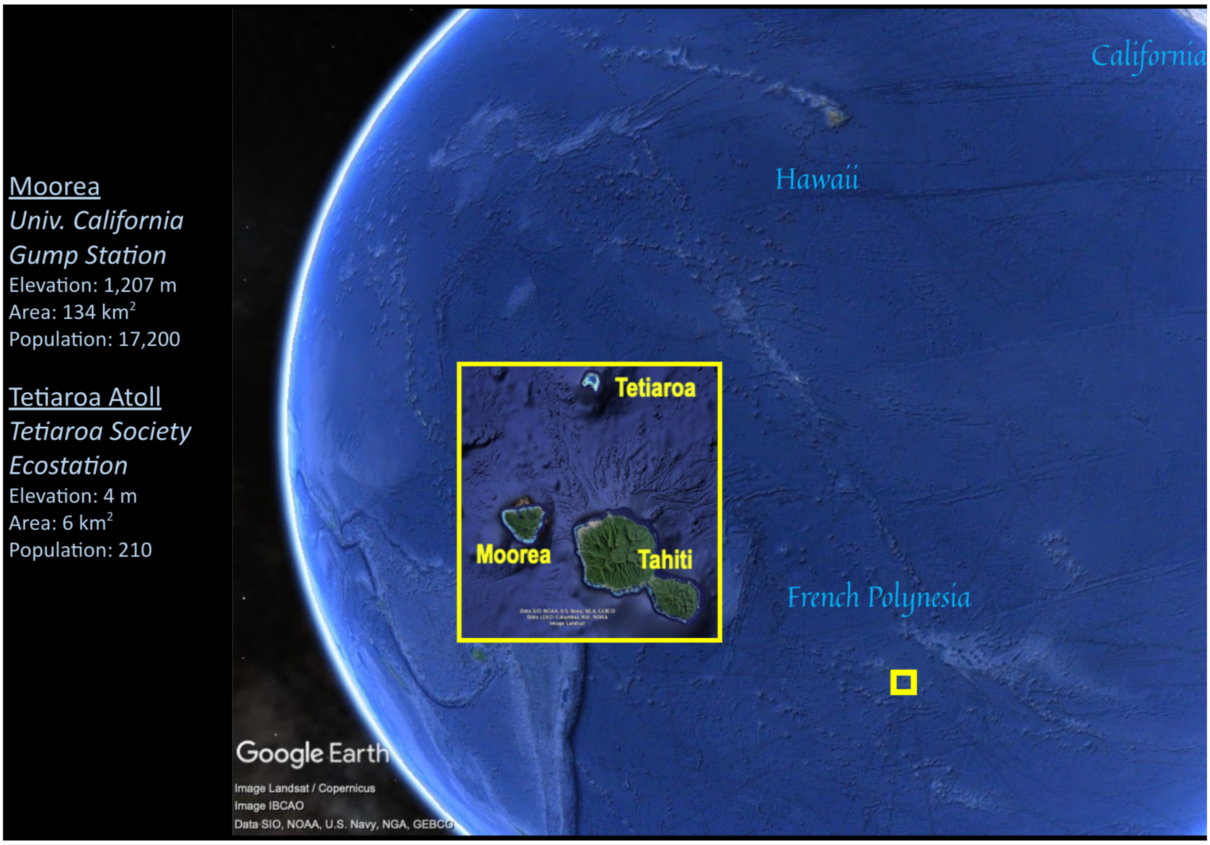

Figure 1. Map of first two field station site locations

FAIR Island Approach

Launched in 2019 with seed funding from the University of California, FAIR Island received additional support from the National Science Foundation (NSF) in August 2021. The project focuses initially on two working field stations - one new and one well-established - that are linked to a range of international research networks in marine and terrestrial environments. Informed by experience at the Gump Station (established 1985), the new international research station (Tetiaroa Ecostation) on the private island of Tetiaroa offers an opportunity to build optimal data infrastructure and practices from the ground up, and to demonstrate the benefits for all stakeholders. Drawing on real use cases, FAIR Island contributes to the advancement and adoption of Open Science at the field stations by building interoperability between existing tools and infrastructure, including Data Management Plans (DMPs), research practice, persistent identifiers (PIDs), data policy, and publications (Figure 2). In addition to a scientific and data-centric focus, the project also addresses ethical, legal, and social issues through application of the CARE Principles [3]. We believe that improving research data infrastructure and practice across these scientific, ethical, legal, and social dimensions at field stations will accelerate place-based research to solve global challenges and benefit local communities (e.g., achieving UN Sustainable Development Goals). Once the FAIR Island infrastructure is established at the initial two stations, the common elements will be shared with other field stations in the Pacific Islands [5], California, and beyond. Through these efforts, we will integrate and test the FAIR and CARE principles addressing whether they have synergies or trade-offs and the extent to which their implementation has measurable impacts on community acceptance, reuse of data, traceability of data products, and rewards/credit for compliance with the principles.

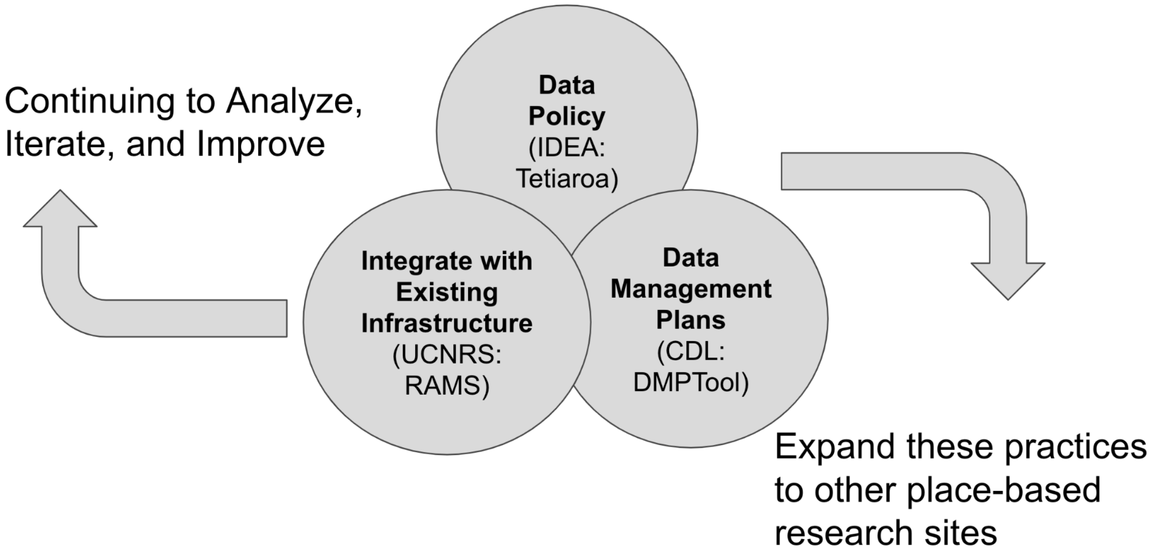

Figure 2: FAIR Island Project diagram

Develop and Test Place-based Research Data Policies

At the time of writing, the Project has collected existing data policy resources, developed a draft Data Policy for the Tetiaroa Ecostation, and solicited initial feedback from the data curation community, including participating in the FAIRsFAIR data policy evaluation [6]. We are now beginning to get feedback from the stakeholders on Tetiaroa starting with Tetiaroa Society's Scientific and Cultural Advisory Boards. Building from the draft Tetiaroa Data Policy, we created and published a Generic Place-Based Data Policy [7] as a template to be shared, refined, and implemented across various field stations and marine labs. By using Github for version control, the development process for this policy is fully transparent, enabling others to easily adopt and adapt it for their use.

Adapting User-Driven Systems Integrations for Networked DMPs

For more than 10 years, DMPs have been included in the proposal phase of the research lifecycle. FAIR Island now extends the use of the DMPs into the implementation phase of research by leveraging the DMPTool to establish a detailed research DMP for work at a field station and mint a DOI for that DMP. For more than 10 years, DMPs have been included in the proposal phase of the research lifecycle. FAIR Island now extends the use of the DMPs into the implementation phase of research by leveraging the DMPTool to establish a detailed research DMP for work at a field station and mint a DOI for that DMP. As research outputs

occur (e.g., data, software, and publications), they are linked to the networked DMP through persistent identifiers (PIDs), creating a hub of activity about a particular research project.

Integration with other systems is a core requirement and scalable benefit of FAIR Island. These PID connections facilitate further development of the DataCite Commons interface and extend connections made possible via the networked DMP. Users can track relationships between DMPs, investigators, outputs, organizations, research methods, and protocols as well as display citations throughout the research lifecycle. A second integration with the UC Natural Reserve System (UCNRS) optimizes the UCNRS reservations management tool (RAMS), which is used at many field stations and provides the initial project metadata needed to create a DMP. This integration reduces the burden on the researcher who need not enter information twice.

As an initial integration experiment, FAIR Island selected the Moorea Biocode Project [8], which ended about 10 years ago. We created a DMP (see https://dmphub.cdlib.org/dmps/doi:10.48321/D1F88S) using the DMPTool with content from the original application submitted to UCNRS RAMS. To find research outputs, the literature was searched for the term ‘Moorea Biocode’. Identified publications and datasets were then linked through related works in the DMP. With these links established, the DataCite DMP ID Jupyter Notebook [9] was used to draw three graphs (Figure 3) that simulate the way that the Persistent Identifier Graph (PID Graph) can visualize relationships across the research lifecycle.

Figure 3: Moorea Biocode PIDGraph Evolution (see https://doi.org/10.5281/zenodo.6347677)

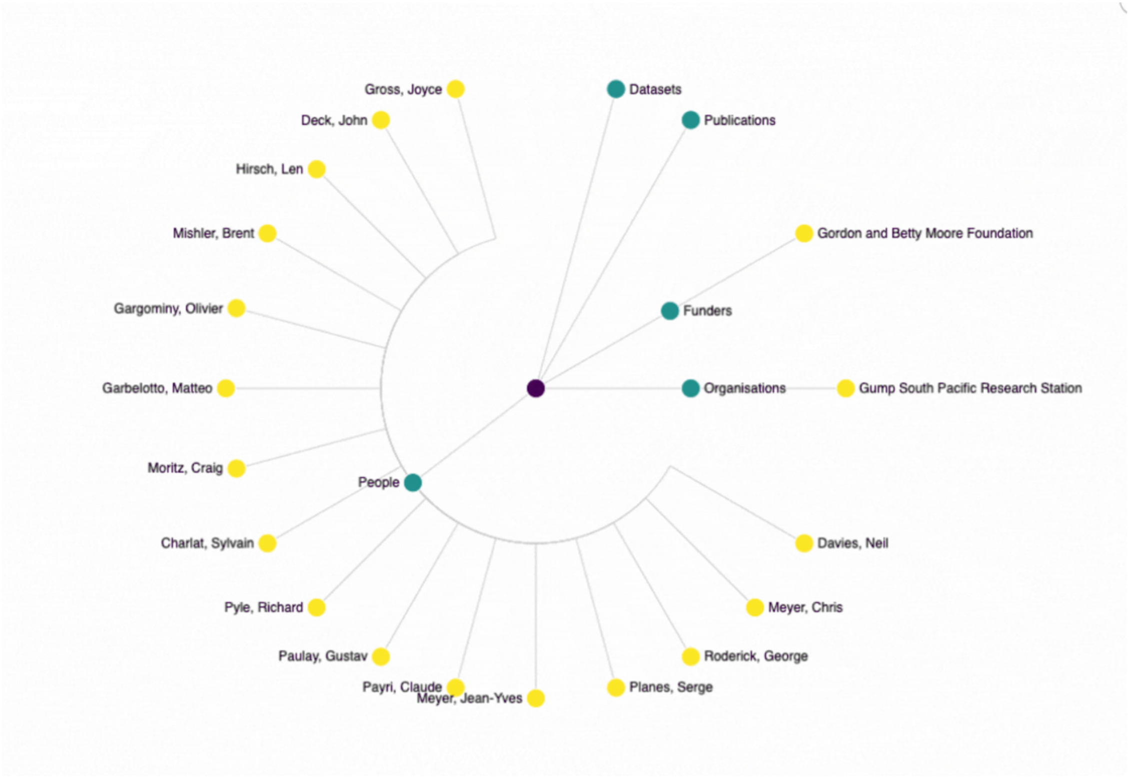

(a) With 'discoverable' people, organizations, and funders before any works were added (other organizations, funders, and individuals could have been involved)

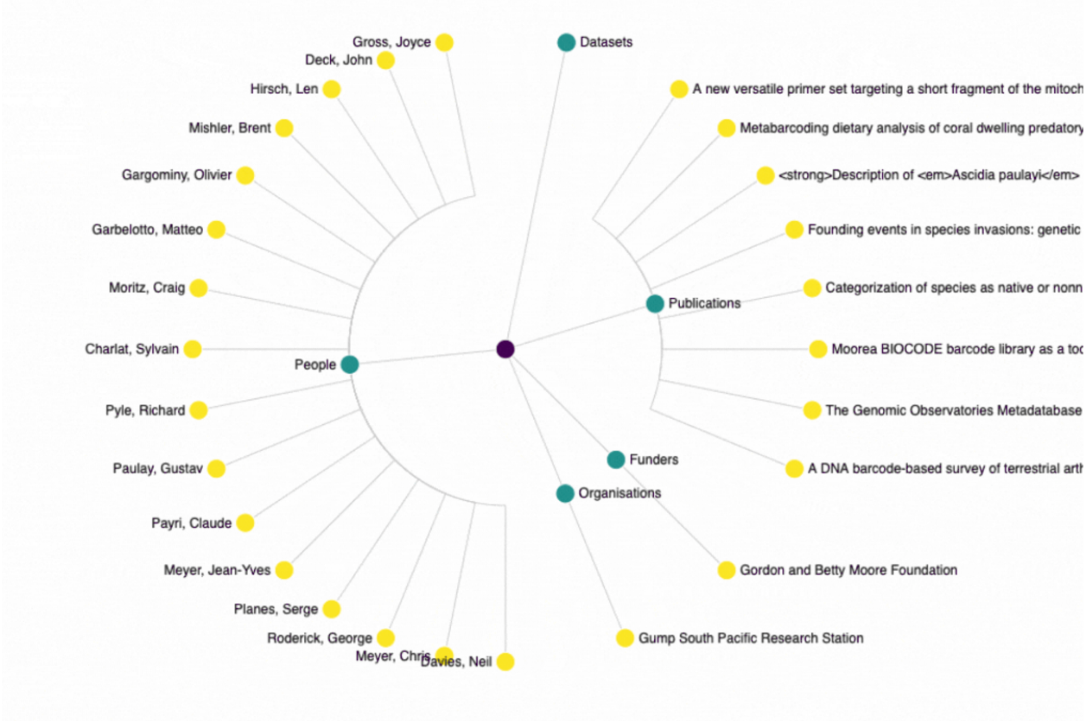

(b) after discoverable journal articles were added

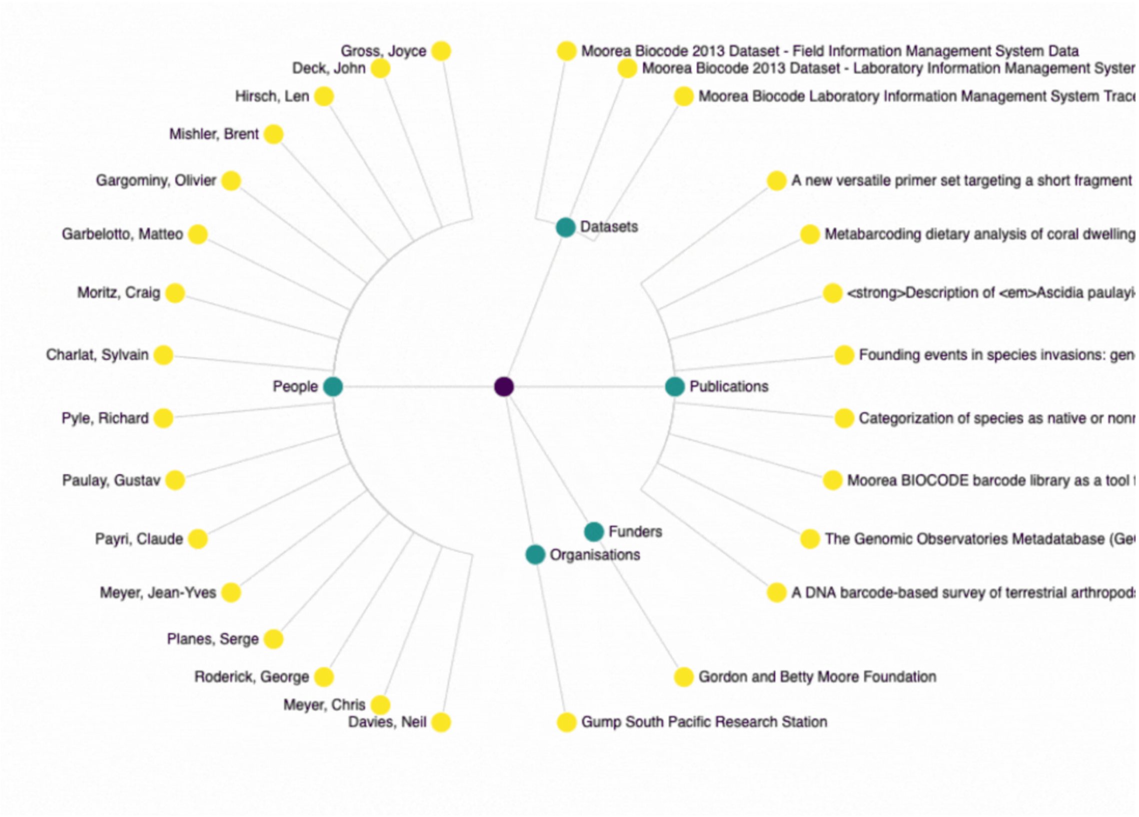

(c) after discoverable datasets were added.

This initial experiment showed the benefits of having structured metadata that included PIDs and could be updated to include scholarly outputs like data and papers. We will ask that each research project have a DMP and if not, we will require using the DMPTool to create one. Moving forward, we are experimenting with minting project DOIs for research projects based on their approved applications. Utilizing the DOI infrastructure will allow for the project metadata to be reused for other aspects of project administration (e.g., permits), reducing the researcher burden. It will also allow for the inclusion of related identifiers, like funding information, DMPs or academic outputs, giving us the benefits found in our initial experiment.

Utilizing this collection of projects with connected outputs, we will develop a field station dashboard showing field data collection to publication, documenting all research data and research outcomes derived from those data. These linkages will provide insight into not only direct connections, but also connections several steps removed, realizing the machine-actionable vision of the FAIR Principles. The understanding that the dashboard will provide an

easier way for field station staff to communicate the value of the work happening at these sites to various stakeholders, including funders and local resource managers. It will also facilitate reuse of data and other research outputs to conduct new science.

Conclusions

The FAIR Island Project is iteratively adjusting to the realities on the ground as it works with real use cases at participating field stations. Each new implementation will allow us to further refine the research infrastructure. The outcomes of this Project will provide insights on optimal research data management practices and how to support place-based research more effectively. The impacts of the Project are likely to be far reaching beyond field stations – to smart cities and other place-based research sites. For more on the Project and to get involved, see the website https://fairisland.org/.

List of Abbreviations

CARE: Collective benefit, Authority to control, Responsibility, Ethics; CDL: California Digital Library; CODATA: Committee on Data for Science and Technology; DMP: Data Management Plan; DOI: Digital Object Identifier; FAIR: Findable, Accessible, Interoperable, Reusable; IDEA: Island Digital Ecosystem Avatars; NSF: National Science Foundation; PID: Persistent Identifier; RAMS: Reservation Application Management System; UCNRS: University of California Natural Reserve System.

Data Availability

Not applicable.

Funding

This material is based upon work supported by the National Science Foundation under "EAGER: The FAIR Island Project for Place-based Open Science" (NSF 2132549) and "RCN: Sampling Nature: A Network to Enhance the Natural History Value Chain for Sustainability Science" (NSF 2129268). Any opinions, findings, and conclusions or recommendations expressed in this material are those of the authors and do not necessarily reflect the views of the National Science Foundation.

Declarations

Not applicable.

Consent for Publication

Not applicable.

Competing Interests

The authors declare that they have no competing interests.

Authors' Contributions

Much of the text is derived from the collaborative "FAIR Island" proposal that was submitted to the National Science Foundation in Spring 2021. E.R. put together the initial draft of the present manuscript with N.D. and all authors contributed to subsequent drafts, with N.D. adding Fig. 1 and E.R. adding Fig. 2, and Fig. 3. All authors read and approved the final manuscript.

Acknowledgments

We are grateful to the initial research teams at the UC Gump South Pacific Research Station and the Tetiaroa Ecostation with whom we have worked.

References

1. CODATA, Committee on Data of the International Science Council, CODATA International Data Policy Committee, CODATA and CODATA China High-level International Meeting on Open Research Data Policy and Practice, Hodson S, Mons B, Uhlir P, et al.. The Beijing Declaration on Research Data. Zenodo. doi: 10.5281/zenodo.3552330

2. Wilkinson MD, Dumontier M, Aalbersberg IJJ, Appleton G, Axton M, Baak A, et al.. The FAIR Guiding Principles for scientific data management and stewardship. Sci Data. 2016; doi: 10.1038/sdata.2016.18.

3. Carroll SR, Garba I, Figueroa-Rodríguez OL, Holbrook J, Lovett R, Materechera S, et al..

The CARE principles for indigenous data governance. Data Sci J. Ubiquity Press, Ltd.; 2020; doi: 10.5334/dsj-2020-043.

4. Davies N, IDEA Consortium, Field D, Gavaghan D, Holbrook SJ, Planes S, et al.. Simulating social-ecological systems: the Island Digital Ecosystem Avatars (IDEA) consortium. GigaScience; doi: 10.1186/s13742-016-0118-5

5. Lemus J, Davies N, Claudet J, Leguen A, Mawyer A, Murphy F, et al.. The 4-Site Pacific Transect Collaborative (4-Site). Mar Technol Soc J. 2021; doi: 10.4031/MTSJ.55.3.47.

6. O'Connor R, Garbuglia F. FAIRsFAIR review of Tetiaroa Research Data Policy. Zenodo; https://doi.org/10.5281/zenodo.6513733

7. Davies N, Chodacki J, Praetzellis M, Nancarrow C, Robinson E. Generic Place-Based Research Data Policy. Zenodo;

8. Check E. Treasure island: pinning down a model ecosystem. Nature. 2006; doi: 10.1038/439378a.

9. Petryszak R, Fenner M, Lambert S, Llinares MB, Madden F. FREYA PID Graph Jupyter Notebooks. DataCite;

10. Robinson. PID Graph for Moorea Biocode Retrospective. Zenodo; doi: 10.5281/zenodo.6347677
