# Supplementary material for: FAIR Island: real-world examples of place-based open science
Source: Gigascience. 2023 Mar 20;12:giad004. doi: 10.1093/gigascience/giad004 (PMC10025936; doi:10.1093/gigascience/giad004)
Supplement: giad004_Response_to_Reviewer_Comments_Original_Submission [file giad004_response_to_reviewer_comments_original_submission.pdf]

Author's Response To Reviewer Comments

Close

We are grateful for the Reviewer's comments.

In response to Reviewer #1 we have have revised the text to introduce the IDEA project earlier.

In response to Reviewer #2:

As requested, we have toned down the rhetoric and revised the text accordingly, indicating our overarching goal rather than presenting a hypothesis to test. We have also modified the Abstract accordingly.

We agree with framing the project as an attempt to test whether the FAIR and CARE principles lead to improvements and we have modified the text to reflect this better.

Further clarification was indeed needed on who we meant by "the community" and we have modified the text to clarify the current status of community feedback and our future intentions.

Close
