# Supplementary material for: FAIR Island: real-world examples of place-based open science
Source: Gigascience. 2023 Mar 20;12:giad004. doi: 10.1093/gigascience/giad004 (PMC10025936; doi:10.1093/gigascience/giad004)
Supplement: giad004_Reviewer_2_Report_Original_Submission — Timothy Clark, Ph.D. -- 12/13/2022 Reviewed [file giad004_reviewer_2_report_original_submission.pdf]

Reviewer Report

Title: FAIR Island: Real-World Examples of Place-based Open Science

Version: Original Submission **Date:** 12/13/2022

Reviewer name: Timothy Clark, Ph.D.

Reviewer Comments to Author:

This Commenary and project overview describes a promising approach to trial implementation of combined FAIR and CARE principles in the context of research stations situated at least partly in indigenous community areas. The approach is meant to take into specific consideration, not only FAIRness, but also ethical respect for the indigenous communities, their cultural practices, and their authoritative control over the data collected about / among them, as defined in the CARE principles.The stated hypothesis is quite grand: "Our hypothesis is that improving research data infrastructure and practice across these scientific, ethical, legal and social dimensions at field stations will accelerate place-based research to solve global challenges ... and benefit local communities". I appreciate the long-term intention this thought expresses, however, as a research hyupothesis, this cannot be evaluated except after many years in a very big study. It should be scaled down here and stated in a form that can be actually evaluated in a proper time-and-place-constrained research study. (I might also suggest dropping the overstated and unnecessary first sentence in the Abstract, which is purely rhetorical, highly debatable, and bears little relationship to what may or may not be demonstrated in the study as it progresses.)Various claims in the paper such as that the proposed approaches should be treated as "best practices" are arguments requiring support. What makes them "best" (or even "good") and according to what criteria? The authors should simply state that they are integrating and testing out two recommended practices, FAIR and CARE, in a certain context, and that their hypothesis is something actually testable, e.g. 1 - *that FAIR and CARE can be appropriately integrated without compromising either principle* and that this will provide 2- * value in the form of some qualitatively or quantitatively evaluable terms *, such as open data, data integration, and community acceptance. Of course the authors must decide what values it is that they are assessing, but that does need to be something that can be declared and assessed. If the hypothesis evaluates as true, in the absence of other results to the contrary, the authors' approach can then be termed (for the time being, as we always seek to improve over time) "best practices", or even better, "recommended practices". Do not overclaim.Another claim made casually in passing is that the authors "solicited feedback from the community". It should be note what form this took, by whom, of whom (who are the community?), with what results, the feedback was solicited and collected. In the actual study when completed, a statistical analysis of the feedback, etc. would be appropriate. CARE principles name "Collective Benefit, Authority to Control, Responsibility" as first three desiderata. "Community feedback" is a surrogate for how "the community" perceives the study will provide these things. At this stage it does need to be stated how this was handled to avoid imprecision - be detailed about what kind of people were queried, what was their relationship to the broader community, how they were solicited, and why that was thought to be a representative sample of opinion.What authors are trying to do here is potentially quite valuable, as claimed in the stated

hypothesis. With a few important modifications, this will be a fine and interesting preliminary report and commentary on the authors' study, which I look forward to reading more about in the future.

Level of Interest

Please indicate how interesting you found the manuscript: Choose an item.

Quality of Written English

Please indicate the quality of language in the manuscript: Choose an item.

Declaration of Competing Interests

Please complete a declaration of competing interests, considering the following questions:

- Have you in the past five years received reimbursements, fees, funding, or salary from an organisation that may in any way gain or lose financially from the publication of this manuscript, either now or in the future?
- Do you hold any stocks or shares in an organisation that may in any way gain or lose financially from the publication of this manuscript, either now or in the future?
- Do you hold or are you currently applying for any patents relating to the content of the manuscript?
- Have you received reimbursements, fees, funding, or salary from an organization that holds or has applied for patents relating to the content of the manuscript?
- Do you have any other financial competing interests?
- Do you have any non-financial competing interests in relation to this paper?

If you can answer no to all of the above, write 'I declare that I have no competing interests' below. If your reply is yes to any, please give details below.

No competing interests

I agree to the open peer review policy of the journal. I understand that my name will be included on my report to the authors and, if the manuscript is accepted for publication, my named report including any attachments I upload will be posted on the website along with the authors' responses. I agree for my report to be made available under an Open Access Creative Commons CC-BY license (http://creativecommons.org/licenses/by/4.0/). I understand that any comments which I do not wish to be included in my named report can be included as confidential comments to the editors, which will not be published.

Choose an item.

To further support our reviewers, we have joined with Publons, where you can gain additional credit to further highlight your hard work (see: https://publons.com/journal/530/gigascience). On publication of this paper, your review will be automatically added to Publons, you can then choose whether or not to claim your Publons credit. I understand this statement.

Yes Choose an item.
